# OA35 Continuing specialist care into adulthood in young people with juvenile idiopathic arthritis: a retrospective cohort study using electronic health records in England

**DOI:** 10.1093/rap/rkac066.035

**Published:** 2022-09-28

**Authors:** Ruth Costello, Lianne Kearsley-Fleet, Janet McDonagh, Kimme Hyrich, Jenny Humphreys

**Affiliations:** London School of Hygiene and Tropical Medicine, London, United Kingdom; University of Manchester, Manchester, United Kingdom; University of Manchester, Manchester, United Kingdom; University of Manchester, Manchester, United Kingdom; University of Manchester, Manchester, United Kingdom

## Abstract

**Introduction/Background:**

It is estimated that juvenile idiopathic arthritis (JIA) persists into adulthood in at least one-third of patients, but it is unclear how frequently hospital services are used as young people move from paediatric to adult specialist services. This study aimed to describe characteristics related to continued specialist care in young people (YP) with JIA using electronic health records (EHR) in England.

**Description/Method:**

YP with JIA were identified from primary care EHR (Clinical Practice Research Datalink GOLD and Aurum databases combined) between 2003 and 2018. JIA was identified if they had a Read code for JIA and either > =3 Hospital Episode Statistics (HES) outpatient specialist care (rheumatology/ophthalmology) appointments or a HES inpatient admission coded with JIA, prior to age 16. Further, cases needed to have linkage to HES data, registration at the same GP for >1 year beyond age 18. Cases were followed from the earliest of first Read code or first HES outpatient appointment until leaving their GP or end of 2018. Continuity of specialist care was categorised into 3 groups by age of last specialist care appointment: prior to 16 years, 16 to 17 years, and 18 years and beyond. YP were considered discharged from specialist care (a proxy for disease remission) if they had > =1 year between the last appointment and end of study follow-up.  Multinomial logistic regression model assessed if any demographic or comorbidity variables were associated with continuing specialist care category.

**Discussion/Results:**

Of 666 YP eligible, 427 (64%) received specialist care beyond age 18, 90 (13%) had their last recorded contact between 16-17 years and 149 (22%) did not continue after 16 years. Patients characteristics are described in Table 1.  Of those continuing beyond 18, 35% (n = 153) were subsequently discharged by study end date. Of all those discharged, 32% had a missed appointment recorded after the last visit attended suggesting failure to attend. In the regression model, female gender and a childhood diagnosis of uveitis were associated with continuing specialist care beyond age 18 compared to those discharged before age 16; respective adjusted odds ratio (95% confidence intervals) 1.84 (1.25, 2.72) and 2.23 (1.14, 4.35).

**Key learning points/Conclusion:**

Over two-third of patients with JIA continue to have hospital visits beyond age 18. Of those, however, approximately one-third were subsequently discharged or lost to follow up from adult specialist care. These data provide important information for YP with JIA and their families, as well as clinicians running, developing and planning young adult services in paediatric and adult rheumatology.

**OA35 Table 1:** Characteristics by continuing specialist care category

		Specialist care until <16 years (n = 149)	Specialist care until 16-17 years (n = 90)	Specialist care ≥ 18 years (n = 427)
Age at first JIA code (%)	*0 - 3 years*	22 (14.8)	6 (6.7)	62 (14.5)
	*4 – 8 years*	43 (28.9)	18 (20.0)	106 (24.8)
	*9 - 15 years*	84 (56.4)	66 (73.3)	259 (60.7)
Gender (%)	*Male*	76 (51.0)	39 (43.3)	159 (37.2)
	*Female*	73 (49.0)	51 (56.7)	268 (62.8)
Discharged (%)		149 (100.0)	90 (100.0)	153 (35.8)
Age at last outpatient appointment attended if discharged (median [IQR])		14.0 [12.8, 15.2]	16.9 [16.5, 17.4]	20.2 [18.7, 22.5]

